# RETRACTION: Comment on ChatGPT and Psoriasis Patient Concerns

**DOI:** 10.1111/srt.70339

**Published:** 2026-02-12

**RETRACTION**: H. Daungsupawong and V. Wiwanitkit, “Comment on ChatGPT and Psoriasis Patient Concerns,” *Skin Research and Technology* 30, no. 5 (2024): e13729, https://doi.org/10.1111/srt.13729.

The above article, published online on 8 May 2024 in Wiley Online Library (wileyonlinelibrary.com), has been retracted by agreement between *Skin Research and Technology*; and John Wiley & Sons Ltd. Following the publication of this Letter to the Editor, a post‐publication assessment revealed that its scientific content lacks substantive value and relevance when compared to the original article object of critique, suggesting that it may have been generated using artificial intelligence tools. Accordingly, the article has been retracted. The authors disagree with this decision.

